# New species and new records of exotic Scolytinae (Coleoptera, Curculionidae) in Europe

**DOI:** 10.3897/BDJ.10.e93995

**Published:** 2022-10-21

**Authors:** Matteo Marchioro, Massimo Faccoli, Marialuisa Dal Cortivo, Manuela Branco, Alain Roques, André Garcia, Enrico Ruzzier

**Affiliations:** 1 Department of Agronomy, Food, Natural Resources, Animals and the Environment (DAFNAE), Legnaro (Padova), Italy Department of Agronomy, Food, Natural Resources, Animals and the Environment (DAFNAE) Legnaro (Padova) Italy; 2 Raggruppamento Carabinieri Biodiversità, Reparto Carabinieri Biodiversità Belluno, Belluno, Italy Raggruppamento Carabinieri Biodiversità, Reparto Carabinieri Biodiversità Belluno Belluno Italy; 3 Forest Research Centre, School of Agriculture, University of Lisbon, Lisboa, Portugal Forest Research Centre, School of Agriculture, University of Lisbon Lisboa Portugal; 4 INRA, UR633 Zoologie Forestière, Orléans, France INRA, UR633 Zoologie Forestière Orléans France

**Keywords:** bark and ambrosia beetles, biological invasions, Coleoptera, exotic species

## Abstract

**Background:**

Bark and ambrosia beetles (Coleoptera, Scolytinae) are amongst the most important wood-boring insects introduced to Europe. During field investigations conducted between 2019 and 2021 in different countries and regions of Europe, many exotic species have been recorded providing new and relevant data.

**New information:**

*Dryoxylononoharaense* (Murayama, 1933) is recorded in Europe for the first time. *Xyleborinusattenuatus* (Blandford, 1894) is a species new to Italy, while *Xylosandrusgermanus* (Blandford, 1894), *Hypothenemuseruditus* (Westwood, 1836) and *Amasa* sp. near *A.truncata* are new country records for Portugal. *Cnestusmutilatus* (Blandford, 1894), *Phloeotribusliminaris* (Harris, 1852) were collected in Italy and *Amasa* sp. near *A.truncata* was collected in France after the first discovery, confirming their establishment and their dispersal into new areas.

## Introduction

Invasive species are one of the major threats to biodiversity, determining substantial negative impacts on forest and agro-ecosystems (Kenis and Branco 2010). The introduction and establishment rate of exotic Scolytinae (Coleoptera, Curculionidae) is globally increasing mostly due to the increment of international trade and climate change (Lantschner et al. 2020, Pureswaran et al. 2022). This trend is expected to further increase despite regulations (Allen et al. 2017), monitoring activities (Rassati et al. 2014, Rabaglia et al. 2019) and the implementation of new early-detection tools and survey methodologies (Poland and Rassati 2018, Marchioro et al. 2020a, Ruzzier et al. 2021a), which, however, may only help to partially reduce the phenomenon.

Kirkendall and Faccoli (2010) provided the first exhaustive review of the exotic species of Scolytinae in Europe, reporting 19 species in the area. Since then, further species have been recorded (Faccoli et al. 2012, Nikulina et al. 2015, Turner and Beaver 2015, Faccoli et al. 2016). Barnouin et al. (2020), while presenting new records of exotic species in France, partially revised some of bark and ambrosia beetles previously introduced to Europe; however, new spreads and detections of new exotic species were recorded in quick succession (Colombari et al. 2022, Gallego et al. 2022, Ruzzier et al. 2022). In addition, some exotic species went through fast expansion phases associated with new introductions, which led them to become almost ubiquitous in most of the European territory (e.g. Galko et al. 2018, Spennemann 2018, Kvamme et al. 2020, Fiala et al. 2021). Most of these exotic species might constitute concrete phytosanitary risks to native forests and arboriculture in general, especially in the case of sudden outbreaks triggered by exceptional weather events, abiotic stressors or the high suitability of the native host-trees colonised by the invaders in the new regions (Ruzzier et al. 2021b, Ranger et al. 2021).

During the years 2019–2021, constant monitoring activities, carried out by the authors as part of biodiversity and invasive species surveys and the monitoring of xylophagous beetles conducted by Reparto Carabinieri Biodiversità Belluno in the forested nature reserves affected by the Vaia storm (Zanella et al. 2020), led to the collection of multiple Scolytine beetles, some of which represent new or relevant faunistic records of exotic species for the European fauna. Here, we present these records.

## Materials and methods

For the morphological identification of the material collected, we used the identification keys provided in Faccoli (2008), Gomez et al. (2018) and Smith et al. (2020). Molecular identification was based on DNA barcodes. DNA extraction, purification and amplification followed the methodology described in Ruzzier et al. (2020). PCR products were purified using Exonuclease and Antarctic Phosphatase (GE Healthcare) and sequenced at the BMR Genomics Service (Padova, Italy). The sequences were edited using MEGA 11 (Tamura et al. 2021) and subsequently, translated with Transeq (EMBOSS) to exclude the presence of stop codons in the coding region. An analysis of the sequences obtained was run through the integrated bioinformatics platform Barcode of Life Data (BOLD) System database to assess the identity of the species. In order to investigate the origin of the Italian *Cnestusmutilatus* population, the single barcode produced by Colombari et al. (2022) was used in a haplotype network analysis in POPART (Leigh and Bryant 2015); as input, we used all the *Cnestus mutilatus* public barcodes available on the BOLD System.

Maps were produced using QGIS 3.16. The basemap originates from the CartoDB Positron, combined with © MapTiler topo and OpenStreetMap data. The reference system of the data is WGA84 - EPSG:4326.

All specimens have been determined by the authors and deposited in the following collections: EDUP - Entomological Collection DAFNAE, Università degli Studi di Padova (Legnaro, Italy); CBPC - Cesare Bellò Private Collection (Castelfranco, Italy); ERPC - Enrico Ruzzier Private Collection (Mirano, Italy); RCBC: Raggruppamento Carabinieri Biodiversità, Reparto Carabinieri Biodiversità (Belluno, Italy); ECIN - INRAE–Zoologie Forestiere Centre de recherche d'Orléans (Orléans, France).

We provide identification remarks only for those species that represent extremely relevant or new European records.

## Taxon treatments

### Dryoxylon onoharaense

(Murayama, 1934)

D7D50293-D464-5D4A-9044-E896CD5ADC86

ON533858

https://www.gbif.org/species/10438835

#### Materials

- **Type status:** Other material. **Occurrence:** recordedBy: G. Cavaletto; individualCount: 1; sex: female; lifeStage: adult; occurrenceID: 61EBDB9A-A3F8-5A42-B552-B37BEE848D1A; **Taxon:** scientificName: *Dryoxylononoharaense* (Murayama, 1934); **Location:** continent: Europe; country: Italy; countryCode: IT; stateProvince: Veneto; county: Padova; decimalLatitude: 45.362254; decimalLongitude: 11.728561; geodeticDatum: WGS84; **Identification:** identifiedBy: Enrico Ruzzier; **Event:** eventDate: 2021-06; **Record Level:** collectionID: ERPC
- **Type status:** Other material. **Occurrence:** recordedBy: G. Cavaletto; individualCount: 1; sex: female; lifeStage: adult; occurrenceID: 14996434-D7BB-5864-860C-2D6A6097CE4E; **Taxon:** scientificName: *Dryoxylononoharaense* (Murayama, 1934); **Location:** continent: Europe; country: Italy; countryCode: IT; stateProvince: Veneto; county: Padova; decimalLatitude: 45.317810; decimalLongitude: 11.703855; geodeticDatum: WGS84; **Identification:** identifiedBy: Enrico Ruzzier; **Event:** eventDate: 2021-06; **Record Level:** collectionID: EDUP
- **Type status:** Other material. **Occurrence:** recordedBy: M. Dal Cortivo; M. Bordin; individualCount: 1; sex: female; lifeStage: adult; occurrenceID: A6CB6693-9DA1-5387-8F6E-47F0FD6A017C; **Taxon:** scientificName: *Dryoxylononoharaense* (Murayama, 1934); **Location:** continent: Europe; country: Italy; countryCode: IT; stateProvince: Veneto; county: Belluno; municipality: Sovramonte; locality: Tavernazzo - R. N. Vette Feltrine; decimalLatitude: 46.091470; decimalLongitude: 11.778480; geodeticDatum: WGS84; **Identification:** identifiedBy: Marialuisa Dal Cortivo; **Event:** eventDate: 2021-07-12; **Record Level:** collectionID: RCBC
- **Type status:** Other material. **Occurrence:** recordedBy: M. Dal Cortivo; M. Bordin; individualCount: 2; sex: female; lifeStage: adult; occurrenceID: 44FB2CF0-9CCD-5493-BD34-605FAEF6CC9E; **Taxon:** scientificName: *Dryoxylononoharaense* (Murayama, 1934); **Location:** continent: Europe; country: Italy; countryCode: IT; stateProvince: Veneto; county: Belluno; municipality: Sovramonte; locality: Tavernazzo - R. N. Vette Feltrine; decimalLatitude: 46.091390; decimalLongitude: 11.777620; geodeticDatum: WGS84; **Identification:** identifiedBy: Marialuisa Dal Cortivo; **Event:** eventDate: 2021-07-12; **Record Level:** collectionID: RCBC
- **Type status:** Other material. **Occurrence:** recordedBy: M. Dal Cortivo; M. Bordin; individualCount: 2; sex: female; lifeStage: adult; occurrenceID: 8D8EC316-7E89-564B-9E5E-275D8802C646; **Taxon:** scientificName: *Dryoxylononoharaense* (Murayama, 1934); **Location:** continent: Europe; country: Italy; countryCode: IT; stateProvince: Veneto; county: Belluno; municipality: Sovramonte; locality: Tavernazzo - R. N. Vette Feltrine; decimalLatitude: 46.091400; decimalLongitude: 11.777360; geodeticDatum: WGS84; **Identification:** identifiedBy: Marialuisa Dal Cortivo; **Event:** eventDate: 2021-07-12; **Record Level:** collectionID: RCBC
- **Type status:** Other material. **Occurrence:** recordedBy: M. Dal Cortivo; M. Bordin; individualCount: 1; sex: female; lifeStage: adult; occurrenceID: F026B69D-1813-5B69-9B20-722618060B68; **Taxon:** scientificName: *Dryoxylononoharaense* (Murayama, 1934); **Location:** continent: Europe; country: Italy; countryCode: IT; stateProvince: Veneto; county: Belluno; municipality: Sovramonte; locality: Tavernazzo - R. N. Vette Feltrine; decimalLatitude: 46.091470; decimalLongitude: 11.778480; geodeticDatum: WGS84; **Identification:** identifiedBy: Marialuisa Dal Cortivo; **Event:** eventDate: 2021-09-01; **Record Level:** collectionID: RCBC

#### Distribution

**Figure 1.**
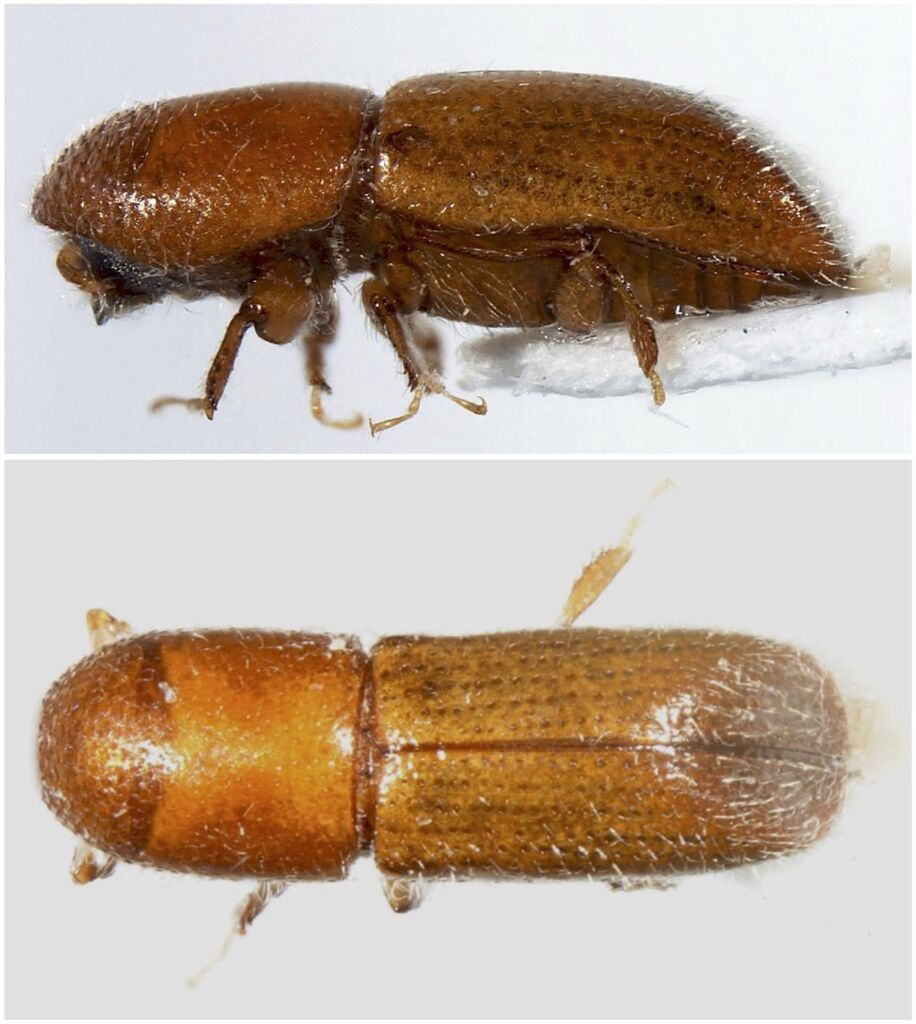
*Dryoxylononoharaense* (Murayama, 1934), female specimen (2.14 mm) from Sovramonte (BL) R.N. Vette Feltrine (Veneto, Italy); lateral view (top), dorsal habitus (bottom) (photocredit: Reparto Carabinieri Biodiversità Belluno).

**Figure 2.**
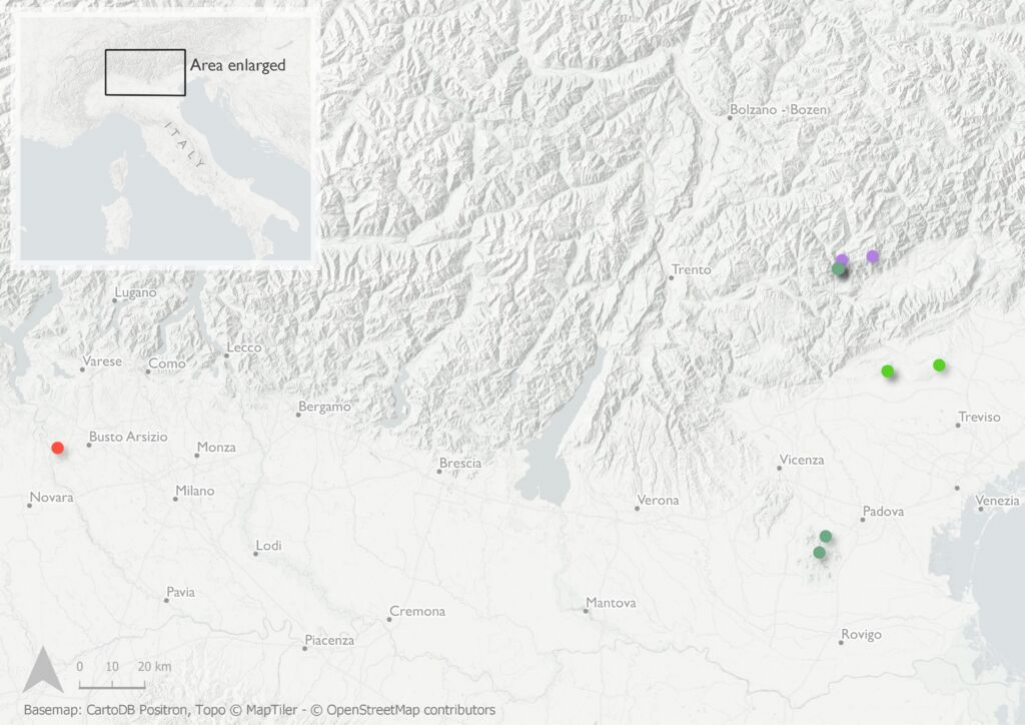
Distribution records of non-native scolytine species in Italy: *Cnestusmutilatus* (Blandford, 1894) [light green]; *Dryoxylononoharaense* (Murayama, 1934) [dark green]; *Phloeotribusliminaris* (Harris, 1852) [red]; *Xyleborinusattenuatus* (Blandford, 1894) [purple].

*Dryoxylononoharaense* (Murayama, 1934) (Fig. 1) is an Eastern Palearctic species belonging to the Xyleborini tribe distributed in China, Japan and South Korea (Smith et al. 2020). Recently, it has been introduced to North America, where it is now widely established (Gomez et al. 2018). The independent collection of eight females in two geographically separated areas in NE Italy indicates the successful establishment of the species. These represent the first records of *D.onoharaense* in the Western Palearctic and a new genus and species to Europe (Fig. 2).

#### Notes

*Dryoxylononoharaense* specimens were collected in the Padua Province (Veneto Region, Italy) by trapping performed in the Euganean hills area using homemade transparent panel traps baited with ethanol. Traps were hung approximately 1 m above the ground, a height where ambrosia beetles are generally abundant (Miller et al. 2019, Marchioro et al. 2020b). The three *D.onoharaense* specimens found from the Belluno Province (Veneto Region, Italy) were collected at Vette Feltrine State Nature Reserve using flight intercept window traps baited with 75% ethanol that were hung from Norway spruce trees in a mixed forest severely damaged by the Vaia storm in 2018.

#### Hosts

The species is polyphagous on broadleaves, recorded from *Acersaccharum* Marshall (Sapindaceae) (Bright and Rabaglia 1999), *Liriodendrontulipifera* L. (Magnoliaceae) (Atkinson 2022), *Populusdeltoides* W.Bartram ex Marshall (Salicaceae) (Coyle et al. 2005) and *Quercus* sp. (Fagaceae) (Murayama 1934); the host plants of this beetle species in Europe remain unknown. Little is known about the biology of *D.onoharaense* and it remains unclear if it is a xylomycetophagous species (Bright and Rabaglia 1999, Bateman et al. 2015); the findings reported in Coyle et al. (2005) suggest myelophagy as a possible feeding habit. The species is included in the European and Mediterranean Plant Protection Organization (EPPO) database (EPPO Code: DRYXON); to date, no direct proof exists regarding any economic or ecological impact of this species.

#### Identification remarks

The identification of the species was confirmed morphologically (using the keys provided in both Gomez et al. (2018) and Smith et al. (2020)), as well as through the DNA barcode (GenBank ref: ON533858) (99.48% of identity on BOLD System).

### Amasa sp.

2ED0A2D4-7070-5722-A569-60C2ABBDACE9

OP143861

OP143862

#### Materials

- **Type status:** Other material. **Occurrence:** individualCount: 5; sex: females; lifeStage: adult; occurrenceID: A4CCA024-9881-5464-976F-7135B03D82F5; **Taxon:** scientificName: *Amasa* sp. near *A.truncata* (Erichson, 1842); **Location:** continent: Europe; country: Portugal; countryCode: PT; county: Lisbon; municipality: Lisbon metropolitan area; decimalLatitude: 38.718138; decimalLongitude: -9.188019; geodeticDatum: WGS84; **Identification:** identifiedBy: Enrico Ruzzier; **Event:** eventDate: 2019-05-03; **Record Level:** collectionID: EDUP
- **Type status:** Other material. **Occurrence:** individualCount: 1; sex: female; lifeStage: adult; occurrenceID: 70D3FF03-4608-5EDD-BFA6-DCE1E4C0EE89; **Taxon:** scientificName: *Amasa* sp. near *A.truncata* (Erichson, 1842); **Location:** continent: Europe; country: Portugal; countryCode: PT; county: Lisbon; municipality: Lisbon metropolitan area; decimalLatitude: 38.70741; decimalLongitude: -9.18294; geodeticDatum: WGS84; **Identification:** identifiedBy: Enrico Ruzzier; **Event:** eventDate: 2019-05-03; **Record Level:** collectionID: EDUP
- **Type status:** Other material. **Occurrence:** individualCount: 1; sex: female; lifeStage: adult; occurrenceID: 3543B123-B333-59D5-988D-3DC641152AAB; **Taxon:** scientificName: *Amasa* sp. near *A.truncata* (Erichson, 1842); **Location:** continent: Europe; country: Portugal; countryCode: PT; county: Lisbon; municipality: Lisbon metropolitan area; decimalLatitude: 38.718138; decimalLongitude: -9.188019; geodeticDatum: WGS84; **Identification:** identifiedBy: Enrico Ruzzier; **Event:** eventDate: 2019-05-24; **Record Level:** collectionID: EDUP
- **Type status:** Other material. **Occurrence:** individualCount: 2; sex: female; lifeStage: adult; occurrenceID: 88526AA3-EDEE-5535-850E-E4B256E2C20D; **Taxon:** scientificName: *Amasa* sp. near *A.truncata* (Erichson, 1842); **Location:** continent: Europe; country: Portugal; countryCode: PT; county: Lisbon; municipality: Lisbon metropolitan area; decimalLatitude: 38.718138; decimalLongitude: -9.188019; geodeticDatum: WGS84; **Identification:** identifiedBy: Enrico Ruzzier; **Event:** eventDate: 2019-06-14; **Record Level:** collectionID: EDUP
- **Type status:** Other material. **Occurrence:** individualCount: 1; sex: female; lifeStage: adult; occurrenceID: CA69BCCD-5A18-5B9A-8219-42A6049912F7; **Taxon:** scientificName: *Amasa* sp. near *A.truncata* (Erichson, 1842); **Location:** continent: Europe; country: Portugal; countryCode: PT; county: Lisbon; municipality: Lisbon metropolitan area; decimalLatitude: 38.718138; decimalLongitude: -9.188019; geodeticDatum: WGS84; **Identification:** identifiedBy: Enrico Ruzzier; **Event:** eventDate: 2019-07-5; **Record Level:** collectionID: EDUP
- **Type status:** Other material. **Occurrence:** individualCount: 2; sex: female; lifeStage: adult; occurrenceID: 0200BAD2-81CE-565E-BE84-888F4ABCB8DE; **Taxon:** scientificName: *Amasa* sp. near *A.truncata* (Erichson, 1842); **Location:** continent: Europe; country: Portugal; countryCode: PT; county: Lisbon; municipality: Lisbon metropolitan area; decimalLatitude: 38.718138; decimalLongitude: -9.188019; geodeticDatum: WGS84; **Identification:** identifiedBy: Enrico Ruzzier; **Event:** eventDate: 2019-07-26; **Record Level:** collectionID: EDUP
- **Type status:** Other material. **Occurrence:** individualCount: 1; sex: female; lifeStage: adult; occurrenceID: 86A2858D-D8C7-5BEE-BC48-9CCF4CDC807C; **Taxon:** scientificName: *Amasa* sp. near *A.truncata* (Erichson, 1842); **Location:** continent: Europe; country: Portugal; countryCode: PT; county: Lisbon; municipality: Lisbon metropolitan area; decimalLatitude: 38.718138; decimalLongitude: -9.188019; geodeticDatum: WGS84; **Identification:** identifiedBy: Enrico Ruzzier; **Event:** eventDate: 2019-08-16; **Record Level:** collectionID: EDUP
- **Type status:** Other material. **Occurrence:** individualCount: 1; sex: female; lifeStage: adult; occurrenceID: 2B4DB422-F06D-54EC-BB5D-EEC4A3FA71E6; **Taxon:** scientificName: *Amasa* sp. near *A.truncata* (Erichson, 1842); **Location:** continent: Europe; country: Portugal; countryCode: PT; county: Lisbon; municipality: Lisbon metropolitan area; decimalLatitude: 38.718138; decimalLongitude: -9.188019; geodeticDatum: WGS84; **Identification:** identifiedBy: Enrico Ruzzier; **Event:** eventDate: 2019-09-06; **Record Level:** collectionID: EDUP
- **Type status:** Other material. **Occurrence:** individualCount: 7; sex: females; lifeStage: adult; occurrenceID: 8B4D6EA8-A01F-55BF-B807-61212945E3A7; **Taxon:** scientificName: *Amasa* sp. near *A.truncata* (Erichson, 1842); **Location:** continent: Europe; country: France; county: Alpes Maritimes; locality: Antibes square Delaunay; decimalLatitude: 43.574134; decimalLongitude: 7.086691; geodeticDatum: WGS84; **Event:** eventDate: 2021; **Record Level:** collectionID: ECIN
- **Type status:** Other material. **Occurrence:** individualCount: 3; sex: females; lifeStage: adult; occurrenceID: CBA7B39A-F88D-5DAA-8830-39C4CFBA9ADD; **Taxon:** scientificName: *Amasa* sp. near *A.truncata* (Erichson, 1842); **Location:** continent: Europe; country: France; county: Alpes Maritimes; locality: Ile Ste Marguerite; decimalLatitude: 43.51772; decimalLongitude: 7.04922; geodeticDatum: WGS84; **Event:** eventDate: 2019; **Record Level:** collectionID: ECIN
- **Type status:** Other material. **Occurrence:** individualCount: 32; sex: females; lifeStage: adult; occurrenceID: 132DF38D-A6D0-59D9-8DBB-315F11859917; **Taxon:** scientificName: *Amasa* sp. near *A.truncata* (Erichson, 1842); **Location:** continent: Europe; country: France; county: Alpes Maritimes; locality: Ile Ste Marguerite; decimalLatitude: 43.51772; decimalLongitude: 7.04922; geodeticDatum: WGS84; **Event:** eventDate: 2020; **Record Level:** collectionID: ECIN
- **Type status:** Other material. **Occurrence:** individualCount: 3; sex: females; lifeStage: adult; occurrenceID: 357863C8-0AE0-5612-9A45-7FC0928F5464; **Taxon:** scientificName: *Amasa* sp. near *A.truncata* (Erichson, 1842); **Location:** continent: Europe; country: France; county: Alpes Maritimes; locality: Ile Ste Marguerite; decimalLatitude: 43.51772; decimalLongitude: 7.04922; geodeticDatum: WGS84; **Event:** eventDate: 2021; **Record Level:** collectionID: ECIN
- **Type status:** Other material. **Occurrence:** individualCount: 1; sex: female; lifeStage: adult; occurrenceID: 15E3DE71-93F4-5BEE-AF91-01BC9E638A8B; **Taxon:** scientificName: *Amasa* sp. near *A.truncata* (Erichson, 1842); **Location:** continent: Europe; country: France; county: Alpes Maritimes; locality: Mandelieu Villa la Desirade; decimalLatitude: 43.546083; decimalLongitude: 6.927778; geodeticDatum: WGS84; **Event:** eventDate: 2021; **Record Level:** collectionID: ECIN
- **Type status:** Other material. **Occurrence:** individualCount: 3; sex: females; lifeStage: adult; occurrenceID: A329E362-2198-5A41-B2F7-7D67BDAD411F; **Taxon:** scientificName: *Amasa* sp. near *A.truncata* (Erichson, 1842); **Location:** continent: Europe; country: France; county: Alpes Maritimes; locality: Théoule/mer- Pointe de l'Aiguille; decimalLatitude: 43.5045901; decimalLongitude: 6.9518406; geodeticDatum: WGS84; **Event:** eventDate: 2021; **Record Level:** collectionID: ECIN
- **Type status:** Other material. **Occurrence:** individualCount: 2; sex: females; lifeStage: adult; occurrenceID: A558F2C7-7BE0-5233-AC33-C1B1BDAC0FBB; **Taxon:** scientificName: *Amasa* sp. near *A.truncata* (Erichson, 1842); **Location:** continent: Europe; country: France; county: Alpes Maritimes; locality: Vallauris- Parc du Paradou; decimalLatitude: 43.560561; decimalLongitude: 7.058094; geodeticDatum: WGS84; **Event:** eventDate: 2020; **Record Level:** collectionID: ECIN
- **Type status:** Other material. **Occurrence:** individualCount: 28; sex: females; lifeStage: adult; occurrenceID: 606BCDB4-461C-570C-A336-55A1BDB8DFA1; **Taxon:** scientificName: *Amasa* sp. near *A.truncata* (Erichson, 1842); **Location:** continent: Europe; country: France; county: Alpes Maritimes; locality: Vallauris- Parc du Paradou; decimalLatitude: 43.560561; decimalLongitude: 7.058094; geodeticDatum: WGS84; **Event:** eventDate: 2021; **Record Level:** collectionID: ECIN
- **Type status:** Other material. **Occurrence:** individualCount: 16; sex: females; lifeStage: adult; occurrenceID: 4BDD6E81-4B30-588A-8A23-627118D256AB; **Taxon:** scientificName: *Amasa* sp. near *A.truncata* (Erichson, 1842); **Location:** continent: Europe; country: France; county: Var; locality: Agay; decimalLatitude: 43.453459; decimalLongitude: 6.865015; geodeticDatum: WGS84; **Event:** eventDate: 2021; **Record Level:** collectionID: ECIN
- **Type status:** Other material. **Occurrence:** individualCount: 1; sex: females; lifeStage: adult; occurrenceID: E9AF28D8-D5F6-568F-88E5-617AF1848570; **Taxon:** scientificName: *Amasa* sp. near *A.truncata* (Erichson, 1842); **Location:** continent: Europe; country: France; county: Var; locality: Le Pradet; decimalLatitude: 43.07991; decimalLongitude: 6.02298; geodeticDatum: WGS84; **Event:** eventDate: 2022; **Record Level:** collectionID: ECIN
- **Type status:** Other material. **Occurrence:** individualCount: 2; sex: females; lifeStage: adult; occurrenceID: 347CE59D-782F-5163-ADF9-FEB7A9FF4DB4; **Taxon:** scientificName: *Amasa* sp. near *A.truncata* (Erichson, 1842); **Location:** continent: Europe; country: France; county: Var; locality: Manjastre- Bormes les Mimosas; decimalLatitude: 43.1622643; decimalLongitude: 6.3114024; geodeticDatum: WGS84; **Event:** eventDate: 2021; **Record Level:** collectionID: ECIN
- **Type status:** Other material. **Occurrence:** individualCount: 1; sex: female; lifeStage: adult; occurrenceID: F84DB6EC-9DD2-56BA-88C0-FDFAC4C113B4; **Taxon:** scientificName: *Amasa* sp. near *A.truncata* (Erichson, 1842); **Location:** continent: Europe; country: France; county: Var; locality: Saint Raphael; decimalLatitude: 43.426722; decimalLongitude: 6.798744; geodeticDatum: WGS84; **Event:** eventDate: 2020; **Record Level:** collectionID: ECIN
- **Type status:** Other material. **Occurrence:** individualCount: 1; sex: female; lifeStage: adult; occurrenceID: 563A0646-0A35-5341-9377-0ABB44E326C2; **Taxon:** scientificName: *Amasa* sp. near *A.truncata* (Erichson, 1842); **Location:** continent: Europe; country: France; county: Bouches du Rhône; locality: Fos/Merl; decimalLatitude: 43.473559; decimalLongitude: 4.861009; geodeticDatum: WGS84; **Event:** eventDate: 2020; **Record Level:** collectionID: ECIN

#### Distribution

The DNA barcode traced back the possible origin of *Amasa* sp. to Australia. This yet unnamed taxon is now present in France, Portugal and possibly Spain (see Barnouin et al. 2020) and it is most probably conspecific with the *Amasa* established in *Eucalyptus* plantations in Brazil, Chile, New Zealand and Uruguay, based on morphological similarity and collecting data (Milligan 1968, Zondag 1977, Flechtmann and Cognato 2011, Gómez et al. 2017, Kirkendall 2018).

#### Notes

**Figure 3.**
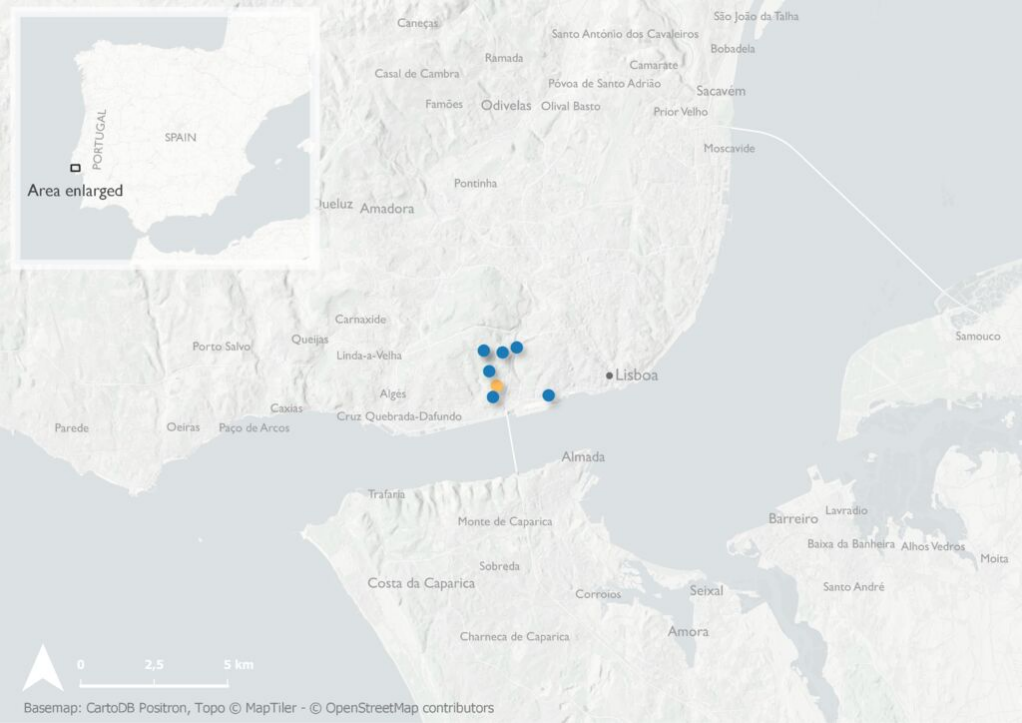
Distribution records of non-native scolytine species in Portugal: *Amasa* sp. near *A.truncata* (Erichson, 1842) [orange]; sites with co-occurence of *Amasa* sp. near *A.truncata* (Erichson, 1842) and *Xylosandrusgermanus* (Blandford, 1894) [blue].

**Figure 4.**
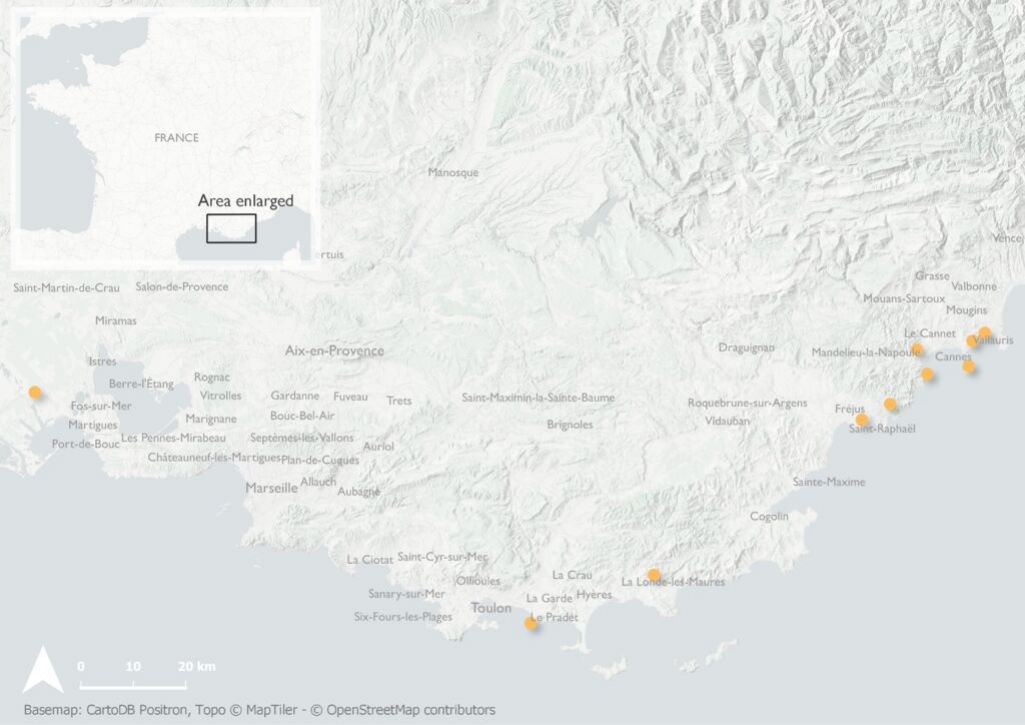
Records of *Amasa* sp. near *A.truncata* (Erichson, 1842) in southern France.

Portuguese *Amasa* specimens were collected in multi-funnel black traps set up at 5 m above the ground and baited with a multi-lure blend of longhorn beetle pheromones, ethanol and alpha-pinene. Traps were located on *Eucalyptus* trees or in their vicinity (Fig. 3). French *Amasa* were collected using multi-funnel black traps baited with either (2018–2020) the 8-pheromone multi-lure blend for cerambycids (Fan et al. 2019) implemented with Ethanol and alpha-pinene or (2021–2022) with four compounds: ethanol, alpha-pinene, alpha-copaene and quercivorol (Fig. 4).

#### Identification remarks

**Figure 5.**
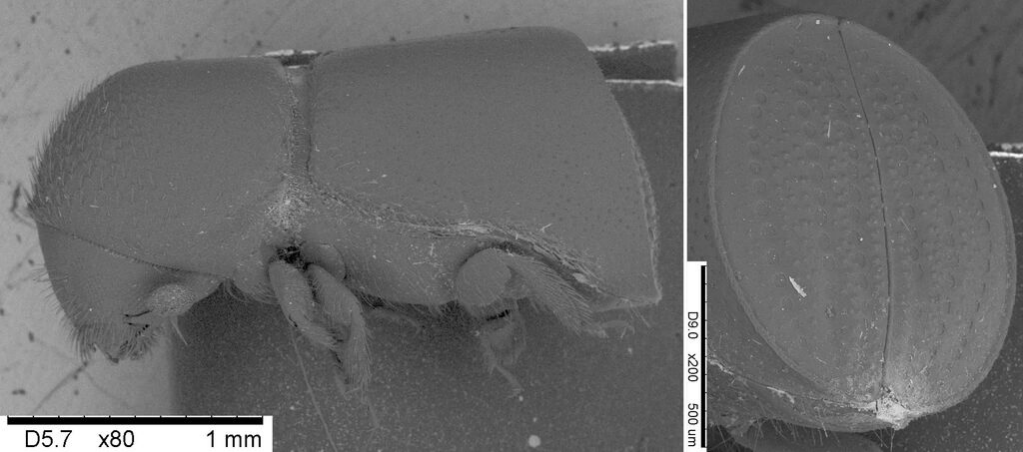
*Amasa* sp. near *A.truncata* (Erichson, 1842), female specimen from Lisbon (Portugal); lateral view (left), postero-lateral view of the elytral declivity (right) (Photocredit: Enrico Ruzzier).

The COI sequences obtained from the specimens collected in Portugal (OP143861 and OP143862), and in France in 2020 and 2021, were identical (100% identity) to the *Amasa* sequence present in the BOLD System (SBGB053-03) (specimen from New South Wales (Australia) and deposited under *Scolytus* sp.) and to those from the specimens collected in 2018 and 2019 in France (Barnouin et al. 2020). As correctly argued by Barnouin et al. (2020), the *Amasa* species recorded in Europe might belong to a single species, still undescribed (Fig. 5).

### Hypothenemus eruditus

(Westwood, 1834)

BC69A2CC-C1BE-5FE6-B24F-E41C3A59861A

https://www.gbif.org/species/7853292

#### Materials

- **Type status:** Other material. **Occurrence:** individualCount: 1; lifeStage: adult; occurrenceID: 0BD15C97-A753-5957-BEAA-DAFAEE72C5B2; **Taxon:** scientificName: *Hypothenemuseruditus* (Westwood, 1834); **Location:** continent: Europe; country: Portugal; countryCode: PT; county: Lisbon; municipality: Lisbon metropolitan area; verbatimLatitude: 38.708401; verbatimLongitude: -9.177198; verbatimCoordinateSystem: WGS84; **Identification:** identifiedBy: Massimo Faccoli; **Event:** eventDate: 2019-05-03; **Record Level:** collectionID: EDUP
- **Type status:** Other material. **Occurrence:** individualCount: 1; lifeStage: adult; occurrenceID: CD63CF04-C7CD-57CC-AC97-2A47773DCFCF; **Taxon:** scientificName: *Hypothenemuseruditus* (Westwood, 1834); **Location:** continent: Europe; country: Portugal; countryCode: PT; county: Lisbon; municipality: Lisbon metropolitan area; verbatimLatitude: 38.711845; verbatimLongitude: -9.185894; verbatimCoordinateSystem: WGS84; **Identification:** identifiedBy: Massimo Faccoli; **Event:** eventDate: 2019-08-16; **Record Level:** collectionID: EDUP

#### Distribution

**Figure 6.**
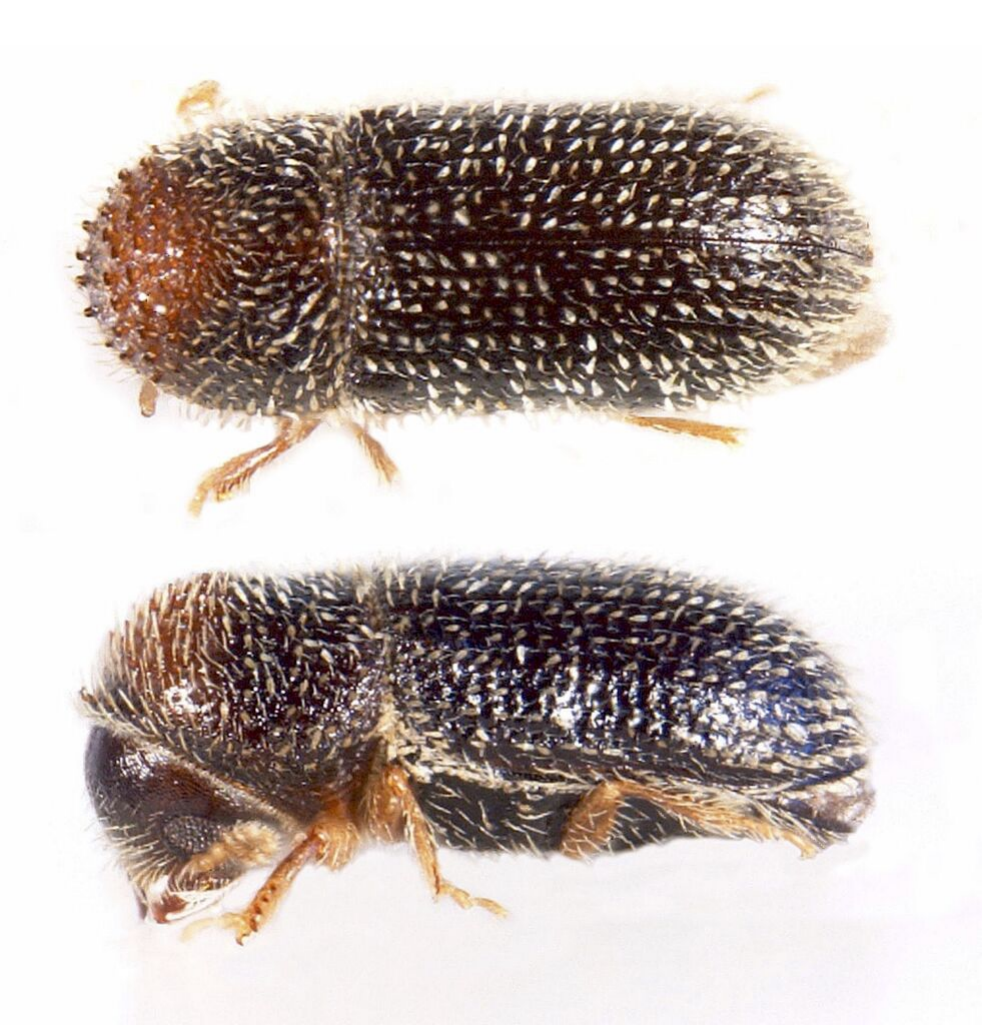
*Hypothenemuseruditus* (Westwood, 1834), female specimen (1.13 mm) from Belluno (Veneto, Italy); lateral view (top), dorsal habitus (bottom) (photocredit: Reparto Carabinieri Biodiversità Belluno).

Cosmopolitan species of tropical and subtropical origin; in Europe, it has been introduced and established in Croatia, France, Italy, Malta, Portugal (Azores), Spain (including the Canary Islands), Russia and Ukraine (Mifsud and Knížek 2009, Knížek 2011). The data provided here represent the first record of the species in continental Portugal (Fig. 6).

#### Notes

All specimens were collected using black multi-funnel traps set up at 5 m above the ground and baited with a multi-lure blend of longhorn beetle pheromones (Fan et al. 2019), ethanol and alpha-pinene.

#### Hosts

An extremely polyphagous species with several hundred host plants, belonging to 81 different families. The most represented hosts are: Anacardiaceae (9 species), Cucurbitaceae (7 species), Euphorbiaceae (14 species), Fabaceae (72 species), Fagaceae (7 species), Juglandaceae (8 species), Malvaceae (24 species), Moraceae (25 species) and Sapindaceae (9 species) (Browne 1961, Schedl 1962, Bright and Skidmore 1997, Bright and Skidmore 2002, Atkinson 2022).

#### Identification remarks

Kambestad et al. (2019) have shown that under the name *eruditus* exists a complex of cryptic species whose identity is not yet defined. Since it is not clear which of these taxa is really present on the European territory, in the present contribution, we refer to *Hypothenemuseruditus* in *sensu lato*.

### Xyleborinus attenuatus

(Blandford, 1894)

F6CEBE0E-394C-5318-A6AC-DD38F69F6961

https://www.gbif.org/species/1178981

#### Materials

- **Type status:** Other material. **Occurrence:** recordedBy: M. Dal Cortivo; M. Bordin; individualCount: 1; sex: female; lifeStage: adult; occurrenceID: 57D5D02E-08E1-5BB1-A108-1AEC3E140929; **Taxon:** scientificName: *Xyleborinusattenuatus* (Blandford, 1894); **Location:** continent: Europe; country: Italy; countryCode: IT; stateProvince: Veneto; county: Belluno; municipality: Sovramonte; locality: Tavernazzo - R. N. Vette Feltrine; decimalLatitude: 46.096750; decimalLongitude: 11.781240; geodeticDatum: WGS84; **Identification:** identifiedBy: Marialuisa Dal Cortivo; **Event:** eventDate: 2021-06-18; **Record Level:** collectionID: RCBC
- **Type status:** Other material. **Occurrence:** recordedBy: M. Dal Cortivo; M. Bordin; individualCount: 1; sex: female; lifeStage: adult; occurrenceID: 91799033-4640-5669-8FAC-5D5EB1EE2ABA; **Taxon:** scientificName: *Xyleborinusattenuatus* (Blandford, 1894); **Location:** continent: Europe; country: Italy; countryCode: IT; stateProvince: Veneto; county: Belluno; municipality: Cesiomaggiore; locality: R.N. Piani Eterni, Erera, Val Falcina: Zoccarè Alto; decimalLatitude: 46.124800; decimalLongitude: 11.911430; geodeticDatum: WGS84; **Identification:** identifiedBy: Marialuisa Dal Cortivo; **Event:** eventDate: 2021-06-18; **Record Level:** collectionID: RCBC

#### Distribution

**Figure 7.**
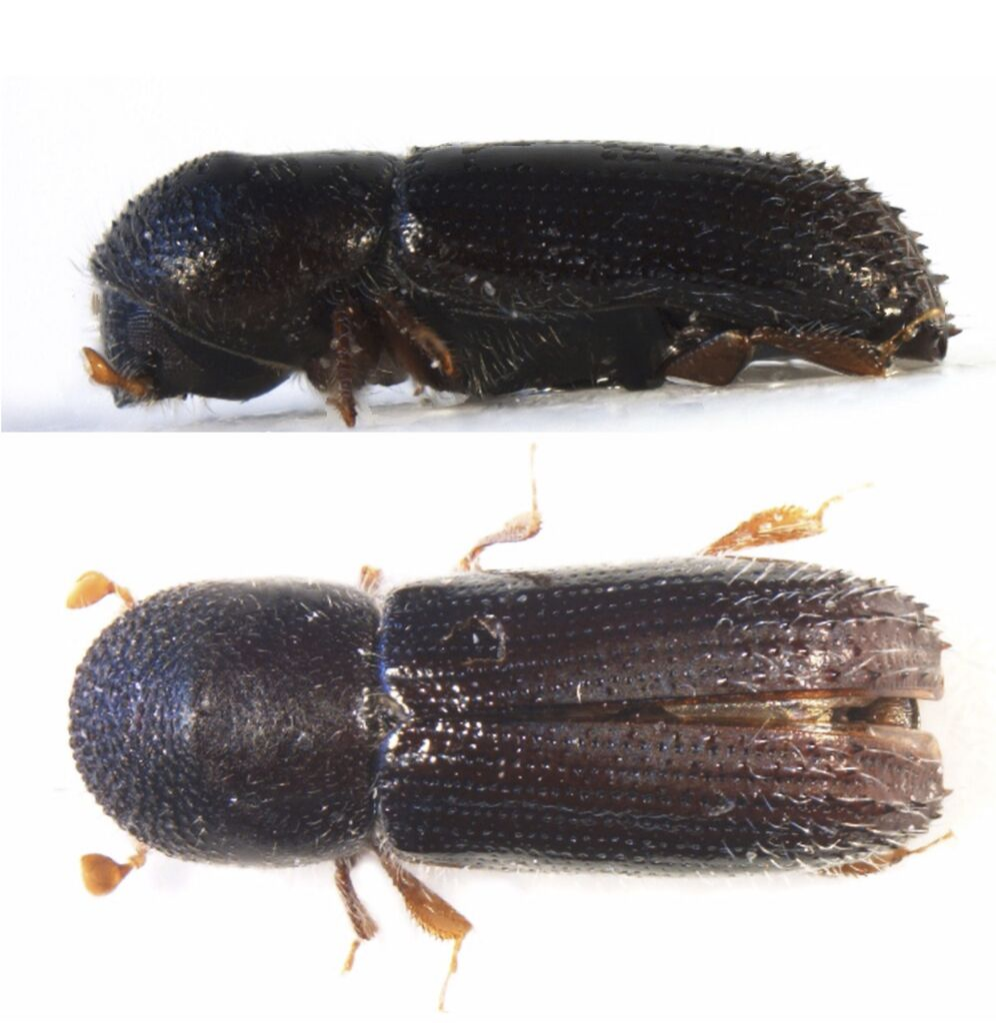
*Xyleborinusattenuatus* (Blandford, 1894), female specimen (2.70 mm) from Sovramonte (BL) R.N. Vette Feltrine (Veneto, Italy); lateral view (top), dorsal habitus (bottom) (photocredit: Reparto Carabinieri Biodiversità Belluno).

A species native of the Eastern Palearctic (China, Japan, Korea, Russia (Far East), Taiwan), introduced and established in most of Europe and North America (Rassati et al. 2016, Kvamme et al. 2020). The data provided here represent the first record of the species in Italy (Fig. 7).

#### Notes

This species was collected using flying intercept window traps baited with 75% ethanol in a mountain beech forest damaged by a wind storm in 2018.

#### Hosts

*Xyleborinusattenuatus* is polyphagous on broadleaves and it was recorded on Betulaceae (10 species), Fagaceae (8 species), Rosaceae (5 species) and Salicaceae (4 species) (Wood and Bright 1992, Bright and Skidmore 1997, Popa et al. 2014). Several recorded host plants are present also in Europe, for example, *Alnusglutinosa* (L.) Gaertn (Betulaceae) (Bright and Skidmore 1997), *Betulapendula* Roth (Betulaceae) (Skrylnik et al. 2019), *Fagussylvatica* L. (Fagaceae) (Sanchez et al. 2020), *Prunusavium* (L.) L. (Rosaceae) (Kvamme et al. 2020), *Quercusrobur* L. (Fagaceae) (Bright and Skidmore 1997), *Sorbusaucuparia* L. (Rosaceae) (Kvamme et al. 2020) and *Fraxinusexcelsior* L. (Oleaceae) (Nikulina et al. 2007).

### Xylosandrus germanus

(Blandford, 1894)

1E4CAE17-769F-5450-BC1C-B5703D1CD2FB

https://www.gbif.org/species/8469824

#### Materials

- **Type status:** Other material. **Occurrence:** individualCount: 1; sex: female; lifeStage: adult; occurrenceID: 4CE4DA07-954B-587E-AF1B-62BAC602173B; **Taxon:** scientificName: *Xylosandrusgermanus* (Blandford, 1894); **Location:** continent: Europe; country: Portugal; countryCode: PT; county: Lisbon; municipality: Lisbon metropolitan area; decimalLatitude: 38.718138; decimalLongitude: -9.188019; geodeticDatum: WGS84; **Identification:** identifiedBy: Massimo Faccoli; **Event:** eventDate: 2019-05-03; **Record Level:** collectionID: EDUP
- **Type status:** Other material. **Occurrence:** individualCount: 1; sex: female; lifeStage: adult; occurrenceID: DF1F8EC3-4D68-5C9D-A963-EDB25B89373F; **Taxon:** scientificName: *Xylosandrusgermanus* (Blandford, 1894); **Location:** continent: Europe; country: Portugal; countryCode: PT; county: Lisbon; municipality: Lisbon metropolitan area; decimalLatitude: 38.719125; decimalLongitude: -9.175109; geodeticDatum: WGS84; **Identification:** identifiedBy: Massimo Faccoli; **Event:** eventDate: 2019-05-03; **Record Level:** collectionID: EDUP
- **Type status:** Other material. **Occurrence:** individualCount: 1; sex: female; lifeStage: adult; occurrenceID: 9D88B2D3-9227-5696-A507-61C444001FAB; **Taxon:** scientificName: *Xylosandrusgermanus* (Blandford, 1894); **Location:** continent: Europe; country: Portugal; countryCode: PT; county: Lisbon; municipality: Lisbon metropolitan area; decimalLatitude: 38.717577; decimalLongitude: -9.180666; geodeticDatum: WGS84; **Identification:** identifiedBy: Massimo Faccoli; **Event:** eventDate: 2019-05-24; **Record Level:** collectionID: EDUP
- **Type status:** Other material. **Occurrence:** individualCount: 1; sex: female; lifeStage: adult; occurrenceID: FDDD0DF0-A97F-5D12-A9F0-F4264DF6FFAF; **Taxon:** scientificName: *Xylosandrusgermanus* (Blandford, 1894); **Location:** continent: Europe; country: Portugal; countryCode: PT; county: Lisbon; municipality: Lisbon metropolitan area; decimalLatitude: 38.703966; decimalLongitude: -9.184431; geodeticDatum: WGS84; **Identification:** identifiedBy: Massimo Faccoli; **Event:** eventDate: 2019-08-16; **Record Level:** collectionID: EDUP
- **Type status:** Other material. **Occurrence:** individualCount: 1; sex: female; lifeStage: adult; occurrenceID: 34666E9D-E862-55AB-B1D7-47492880CBC1; **Taxon:** scientificName: *Xylosandrusgermanus* (Blandford, 1894); **Location:** continent: Europe; country: Portugal; countryCode: PT; county: Lisbon; municipality: Lisbon metropolitan area; decimalLatitude: 38.704480; decimalLongitude: -9.162623; geodeticDatum: WGS84; **Identification:** identifiedBy: Massimo Faccoli; **Event:** eventDate: 2019-08-16; **Record Level:** collectionID: EDUP
- **Type status:** Other material. **Occurrence:** individualCount: 1; sex: female; lifeStage: adult; occurrenceID: 619CA317-0C4C-5553-B77A-45C4F3B9E116; **Taxon:** scientificName: *Xylosandrusgermanus* (Blandford, 1894); **Location:** continent: Europe; country: Portugal; countryCode: PT; county: Lisbon; municipality: Lisbon metropolitan area; decimalLatitude: 38.711845; decimalLongitude: -9.185894; geodeticDatum: WGS84; **Identification:** identifiedBy: Massimo Faccoli; **Event:** eventDate: 2019-09-30; **Record Level:** collectionID: EDUP

#### Distribution

**Figure 8.**
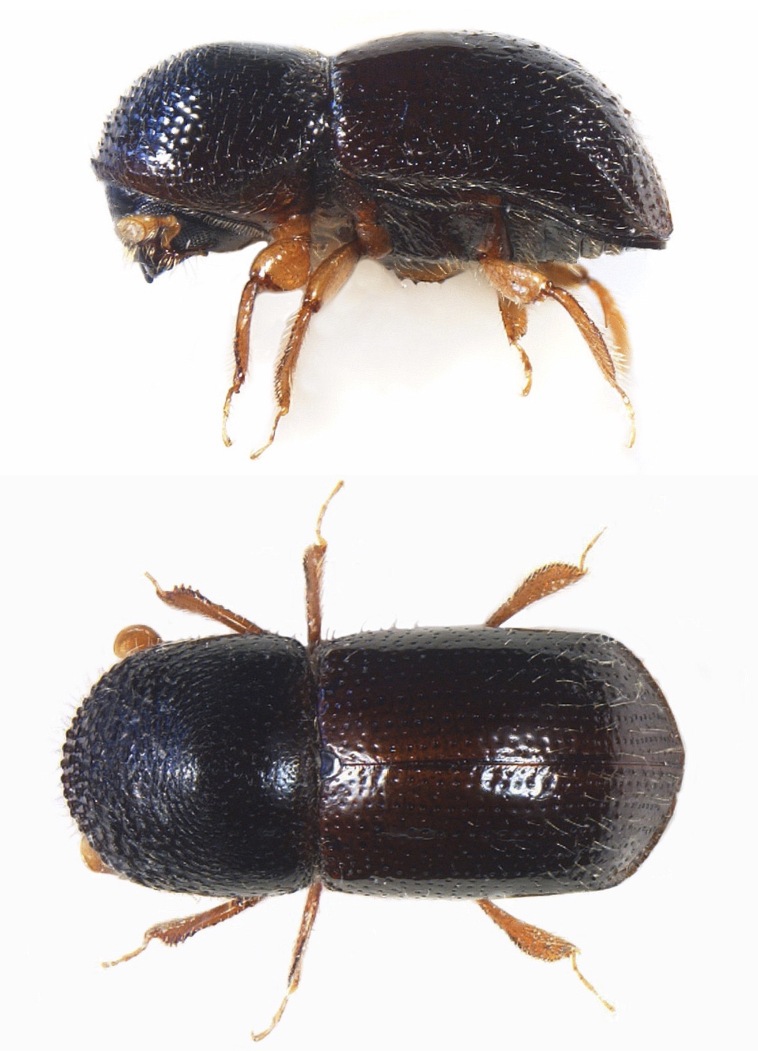
*Xylosandrusgermanus* (Blandford, 1894), female specimen (2.20 mm) from Belluno (Veneto, Italy); lateral view (top), dorsal habitus (bottom) (photocredit: Reparto Carabinieri Biodiversità Belluno).

Species native of the Oriental Region and Eastern Palearctic (Smith et al. 2020), introduced and widely established in Europe and North America (Galko et al. 2018, Gomez et al. 2018). The data provided here represent the first record of the species in Portugal (Fig. 8). This represents the westernmost location in Europe.

#### Notes

All specimens were collected using black multi-funnel traps set up at 5 m above the ground and baited with a multi-lure blend of longhorn beetle pheromones (Fan et al. 2019), ethanol and alpha-pinene.

#### Hosts

Extremely polyphagous species, with hundreds of host plants recorded. The most represented families are: Anacardiaceae (11 species), Betulaceae (17 species), Fabaceae (7 species), Fagaceae (26 species), Juglandaceae (9 species), Lauraceae (18 species), Rosaceae (20 species), Sapindaceae (15 species) and Ulmaceae (7 species) (Murayama 1953, Weber and McPherson 1983, Dole and Cognato 2010).

### Cnestus mutilatus

(Blandford, 1894)

8CD7E56C-62E0-5741-ABAB-DFF3B70B7555

https://www.gbif.org/species/6132425

#### Materials

- **Type status:** Other material. **Occurrence:** recordedBy: C. Bellò, M. Attorino; individualCount: 2; sex: females; lifeStage: adult; occurrenceID: 6FD1A1F1-FA44-5C1A-97AF-BED03D0743E6; **Taxon:** scientificName: *Cnestusmutilatus* (Blandford, 1894); **Location:** continent: Europe; country: Italy; countryCode: IT; stateProvince: Veneto; county: Treviso; municipality: Maser; locality: strada per Forcella Moscaccin; decimalLatitude: 45.813500; decimalLongitude: 11.970306; geodeticDatum: WGS84; **Identification:** identifiedBy: Enrico Ruzzier; **Event:** eventTime: 2022-02-03; **Record Level:** collectionID: CBPC
- **Type status:** Other material. **Occurrence:** recordedBy: M. Marchioro; individualCount: 2; sex: females; lifeStage: adult; occurrenceID: FAB6D080-EA94-535C-BBFF-04FCEA3B4024; **Taxon:** scientificName: *Cnestusmutilatus* (Blandford, 1894); **Location:** continent: Europe; country: Italy; countryCode: IT; stateProvince: Veneto; county: Treviso; municipality: Nervesa della Battaglia; locality: bosco del Montello; decimalLatitude: 45.829772; decimalLongitude: 12.171981; geodeticDatum: WGS84; **Identification:** identifiedBy: Enrico Ruzzier; **Event:** eventTime: 2021-08-15; **Record Level:** collectionID: EDUP
- **Type status:** Other material. **Occurrence:** recordedBy: M. Marchioro; individualCount: 2; sex: females; lifeStage: adult; occurrenceID: 73D8A4F2-4D98-57B8-9A40-D16F7C9DB7AA; **Taxon:** scientificName: *Cnestusmutilatus* (Blandford, 1894); **Location:** continent: Europe; country: Italy; countryCode: IT; stateProvince: Veneto; county: Treviso; municipality: Nervesa della Battaglia; locality: bosco del Montello; decimalLatitude: 45.813500; decimalLongitude: 11.970306; geodeticDatum: WGS84; **Identification:** identifiedBy: Enrico Ruzzier; **Event:** eventTime: 2021-08-15; **Record Level:** collectionID: EDUP

#### Distribution

This species, native to the Oriental and Eastern Palearctic Regions, is now introduced and established in North America (Gomez et al. 2018, Smith et al. 2020). *Cnestusmutilatus* has been recently recorded in Europe (Italy) on the basis of a single specimen collected in NE Italy (Colombari et al. 2022). The discovery of the species in two sites located at about 20 km from the record mentioned above clearly indicates its establishment in the Veneto Region (NE Italy)(Fig. 2)

#### Notes

**Figure 9.**
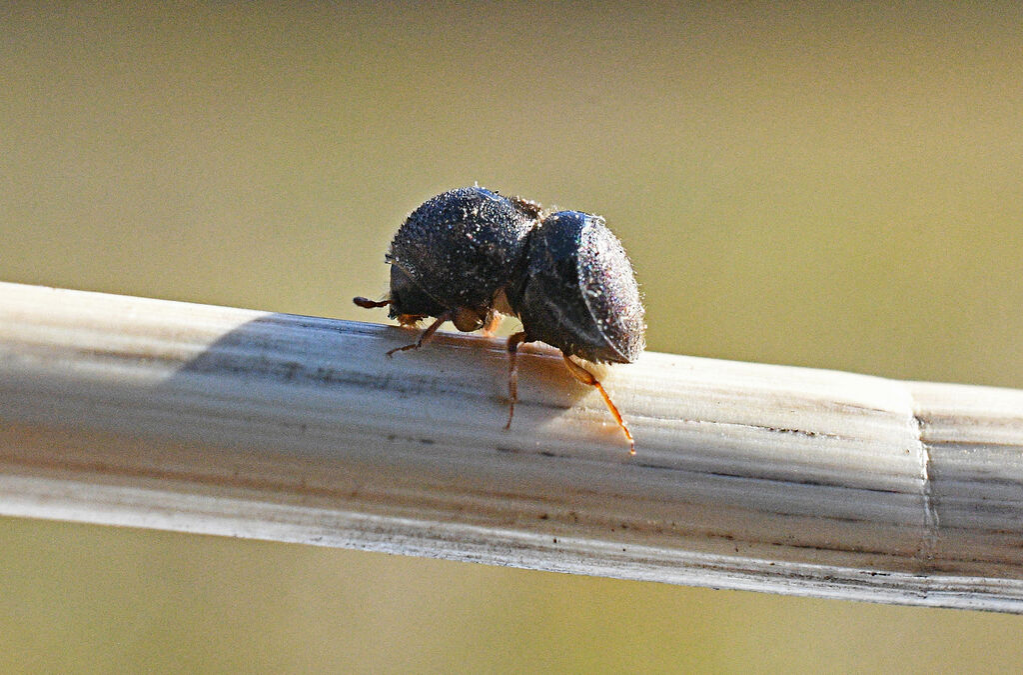
*Cnestusmutilatus* collected in Maser (February 2022), photographed in nature (photocredit: Pietro Berton).

*Cnestusmutilatus* from Maser (“strada per forcella Moscaccin”, Treviso -Italy) was collected by sifting forest litter under *Quercus* sp. during wintertime (*Fig. 9*). The other specimens were collected during a survey conducted in Veneto Region using black multi-funnel traps baited with ethanol and alpha-pinene.

#### Hosts

For *C.mutilatus*, more than forty host plant species have been recorded, mostly belonging to Fabaceae (5 species), Fagaceae (4 species), Lauraceae (7 species) and Sapindaceae (5 species) (Wood and Bright 1992, Mandelshtam et al. 2018, Smith et al. 2020). Amongst the reported host plants, some are also present in Europe and have economic significance (*i.e. Juglans regia* L. (Juglandaceae), *Morusalba* L. (Moraceae), *Prunusserotina* Ehrh (Rosaceae) and *Vitisrotundifolia* Michx. (Vitaceae)) (Mandelshtam et al. 2018, Smith et al. 2020, Ruzzier et al. 2021b).

#### Identification remarks

**Figure 10.**
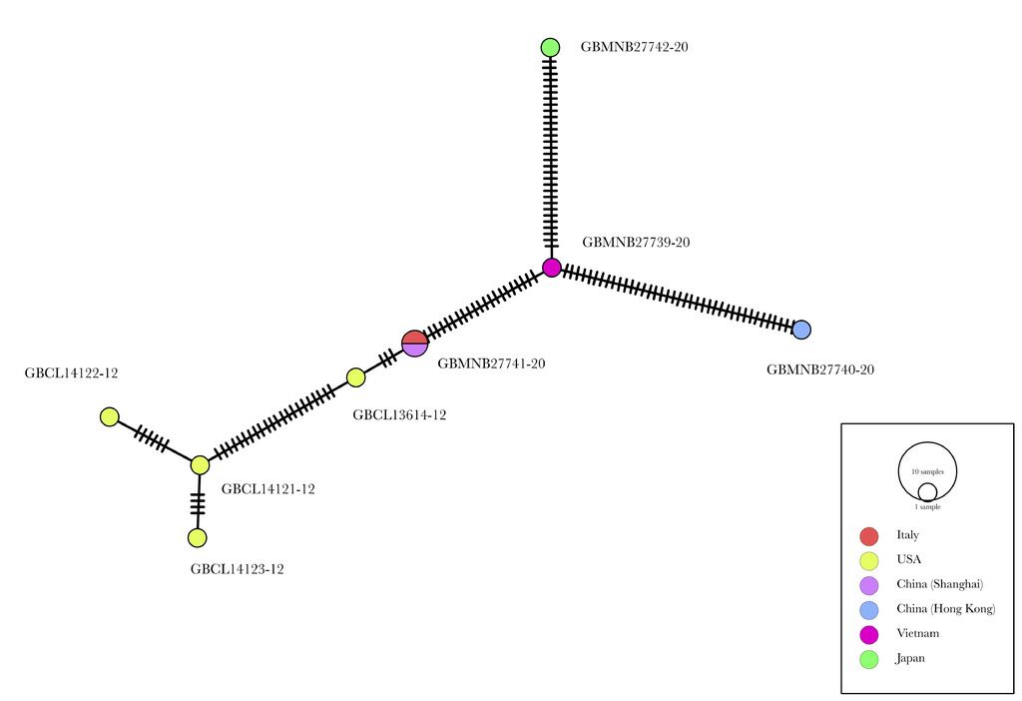
Haplotype network of *Cnestusmutilatus* COI sequences available on BOLD Systems; network constructed using the Minimum Spanning Network approach (image credit: Enrico Ruzzier).

The haplotype network indicates the Eastern Palaearctic origin of the *C.mutilatus* Italian population; in particular, the 100% identity between the Italian GBMNF53732-22, sequenced by Colombari et al. (2022) and GBMNB27741-2, sequenced by Cognato et al. (2020), indicates Shanghai (China) as the possible point of origin (Fig. 10).

### Phloeotribus liminaris

(Harris, 1852)

2A42442F-3E75-528A-BCA1-6093B360B2B4

#### Materials

- **Type status:** Other material. **Occurrence:** recordedBy: A. Galli; individualCount: 1; sex: male; lifeStage: adult; occurrenceID: EC8EEA63-30C8-56DC-8390-FA175BC61ADB; **Taxon:** scientificName: *Phloeotribusliminaris* (Harris, 1852); **Location:** continent: Europe; country: Italy; countryCode: IT; stateProvince: Lombardy; county: Varese; municipality: Linate Pozzolo; decimalLatitude: 45.604167; decimalLongitude: 8.729500; geodeticDatum: WGS84; **Identification:** identifiedBy: Enrico Ruzzier; **Event:** eventTime: 2021-05-03; **Record Level:** collectionID: ERPC

#### Distribution

**Figure 11.**
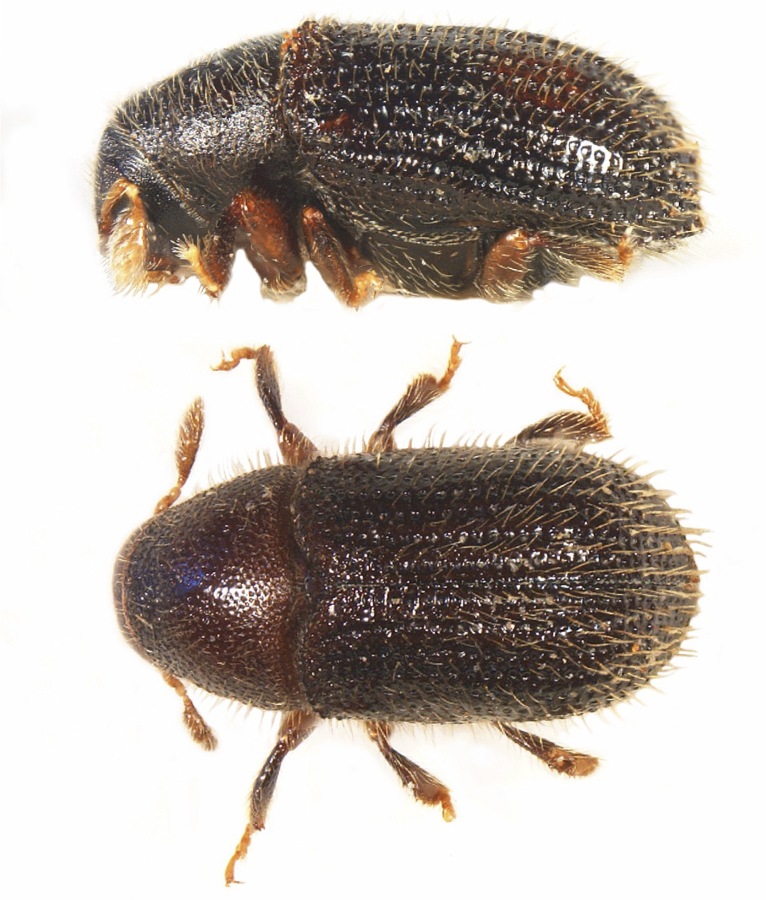
*Phloeotribusliminaris* (Harris, 1852), male specimen (2.08 mm) from Ticino Park (Lombardy, Italy); lateral view (top), dorsal habitus (bottom) (photocredit: Reparto Carabinieri Biodiversità Belluno).

Species of Nearctic origin was recorded for the first time in Europe in Lombardy (North Italy) in 2003 (Pennacchio et al. 2004). The species seems to present a very limited dispersal capability, not having substantially expanded its distribution range in nearly 10 years. The establishment of *P.liminaris* in Italy is confirmed by the collection of this single male specimen (Fig. 2; Fig. 11). *Phloeotribusliminaris* has also been recently intercepted in France, where, however, it is not naturalised (Barnouin et al. 2020).

#### Notes

*Phloeotribusliminaris* was captured using bottle traps baited with red wine and placed at about 2.5 m from the ground (see Ruzzier et al. 2021a).

#### Hosts

Despite the species being considered of potential phytosanitary interest for Mediterranean *Prunus* spp. (Rosaceae) (Pennacchio et al. 2004), to date, no ecological or economic impact caused by *Phloeotribusliminaris* has ever been recorded in Italy.

## Discussion

The records presented here show once again how Europe and especially circum-Mediterranean countries are extremely prone to biological invasions by exotic species of possible forest and phytosanitary interest. Despite the adoption of strict international regulations and newly-implemented detection strategies, the number of exotic Scolytine species continuously and quickly increases year by year. Italy is the country with the highest number of exotic coleopteran species in Europe, as already recorded in Molfini et al. (2020), Ruzzier et al. (2020a), Ruzzier et al. (2021c) and Domina (2021), most plausibly because of the high habitat diversity of Italian ecosystems and the central role of Italian ports in international trade (Rassati et al. 2013). Furthermore, in association with the natural spread of the species, national trade and unregulated movement of goods within the European Member States might have favoured and boosted the dispers althrough the EU of highly-adaptable species, such as *Xyleborinusattenuatus*, *Xylosandrusgermanus* and *Hypothenemuseruditus*. Considering the changes of the Scolytinae exotic fauna recorded in Europe since Kirkendall and Faccoli (2010), we can observe a constant homogenisation in the composition of exotic species between North America and Europe. The biological invasions involve, in fact, almost the same species, most of which have Eastern Palearctic or Oriental origins. Such a condition suggests that the arrival of new exotic Scolytinae in Europe might happen not only via a direct introduction from their native areas, but also via indirect introductions from previously-invaded regions. However, it remains to be understood how the exotic species documented in the last few years in Europe have been able to elude any detection at entry points, acclimatise in nature and spread so rapidly.

## Supplementary Material

XML Treatment for Dryoxylon onoharaense

XML Treatment for Amasa sp.

XML Treatment for Hypothenemus eruditus

XML Treatment for Xyleborinus attenuatus

XML Treatment for Xylosandrus germanus

XML Treatment for Cnestus mutilatus

XML Treatment for Phloeotribus liminaris
